# Confocal Laser Endomicroscopy in Gastrointestinal and Pancreatobiliary Diseases: A Systematic Review and Meta-Analysis

**DOI:** 10.1155/2016/4638683

**Published:** 2016-02-17

**Authors:** Alessandro Fugazza, Federica Gaiani, Maria Clotilde Carra, Francesco Brunetti, Michaël Lévy, Iradj Sobhani, Daniel Azoulay, Fausto Catena, Gian Luigi de'Angelis, Nicola de'Angelis

**Affiliations:** ^1^Unit of Gastroenterology and Digestive Endoscopy, University of Parma, 43100 Parma, Italy; ^2^University Paris VII-Denis Diderot, 75006 Paris, France; ^3^Unit of Digestive, Hepato-Pancreato-Biliary Surgery and Liver Transplantation, Henri Mondor Hospital, AP-HP, 94010 Créteil, France; ^4^Department of Gastroenterology and Digestive Endoscopy, Henri Mondor Hospital, AP-HP, 94010 Créteil, France; ^5^Cancer Research Lab. EC2M3, Université Paris-Est, Val de Marne UPEC, 94010 Créteil, France; ^6^Emergency Surgery Department, University of Parma, 43100 Parma, Italy; ^7^Department of Advanced Biomedical Sciences, University Federico II of Naples, 80138 Naples, Italy

## Abstract

Confocal laser endomicroscopy (CLE) is an endoscopic-assisted technique developed to obtain histopathological diagnoses of gastrointestinal and pancreatobiliary diseases in real time. The objective of this systematic review is to analyze the current literature on CLE and to evaluate the applicability and diagnostic yield of CLE in patients with gastrointestinal and pancreatobiliary diseases. A literature search was performed on MEDLINE, EMBASE, Scopus, and Cochrane Oral Health Group Specialized Register, using pertinent keywords without time limitations. Both prospective and retrospective clinical studies that evaluated the sensitivity, specificity, or accuracy of CLE were eligible for inclusion. Of 662 articles identified, 102 studies were included in the systematic review. The studies were conducted between 2004 and 2015 in 16 different countries. CLE demonstrated high sensitivity and specificity in the detection of dysplasia in Barrett's esophagus, gastric neoplasms and polyps, colorectal cancers in inflammatory bowel disease, malignant pancreatobiliary strictures, and pancreatic cysts. Although CLE has several promising applications, its use has been limited by its low availability, high cost, and need of specific operator training. Further clinical trials with a particular focus on cost-effectiveness and medicoeconomic analyses, as well as standardized institutional training, are advocated to implement CLE in routine clinical practice.

## 1. Introduction

Confocal laser endomicroscopy (CLE) is an endoscopic modality that was developed to obtain very high magnification of the mucosal layer of the gastrointestinal (GI) tract [1], and it has the potential to enable histological diagnosis in real time [2]. CLE is based on tissue illumination using a low-power laser and the subsequent detection of fluorescent light that is reflected back from the tissue through a pinhole [3]. The term “confocal” refers to the alignment of both illumination and collection systems in the same focal plane [4, 5]. In brief, the laser light is focused at a selected depth in the tissue of interest and reflected light is then refocused onto the detection system by the same lens. Only the returning light that is refocused through the pinhole is detected. Any light that is reflected or scattered at other geometric angles from the illuminated object or any light that is refocused out of plane with the pinhole is excluded from detection. This dramatically increases the spatial resolution of CLE and enables cellular imaging and evaluation of tissue architecture at the focal plane to be performed during endoscopy [1].

To obtain confocal images, exogenous fluorescence agents can be administered either topically or systemically [6]. The most common topical contrast agents that are applied by a spraying catheter are acriflavine and cresyl violet [7], whereas the most widely used systemically administered fluorescent agent is intravenous fluorescein sodium. Fluoresceins are nontoxic, and their administration has been associated with only rare adverse events, including transient hypotension without shock (0.5%), nausea (0.39%), injection site erythema (0.35%), self-limited diffuse rash (0.04%), and mild epigastric pain (0.09%) [8].

There are two types of CLE: endoscope-based (eCLE) and probe-based (pCLE) endomicroscopy. To perform eCLE, a dedicated endoscope with a miniaturized confocal scanner integrated into the distal tip is employed [9]. This system was developed by Pentax (Tokyo, Japan) and enables simultaneous endoscopic imaging to be performed. Additionally, it frees the endoscopic working channel, and it can be used for targeted biopsies or combined enhancement techniques [9, 10]. Images can be collected by sectioning down through the mucosa in 7 *μ*m increments to a depth of 250 *μ*m. Image stabilization is necessary to obtain good quality images [11]. The second system is pCLE (Cellvizio, Mauna Kea Technologies, Paris, France). In this system, confocal miniprobes can be passed down the accessory channel of any standard endoscope [12], providing rapid image capture and “stitching” of adjacent images to create a “mosaic image” in real time [11]. The advantages of pCLE are its versatility and the possibility of combining it with other imaging modalities such as virtual chromoendoscopy or magnification [9]. Image stabilization can be achieved by using a plastic cap on the endoscope tip. There are several types of probes that are characterized by different imaging depths, fields of view, and lateral resolutions (Supplementary Table S1 in Supplementary Material available online at http://dx.doi.org/10.1155/2016/4638683). More recently, a novel needle-based CLE (nCLE) miniprobe (AQ-Flex 19; Mauna Kea) has been developed which can pass through a 19G needle to perform endoscopic ultrasound-guided fine-needle aspiration (EUS–FNA) on solid organs and lymph nodes [13–16]. The probes can be reused after disinfection for as many as 10 to 20 examinations [1].

CLE can be applied to examine luminal structures, such as esophagus, stomach, and colon, and ductal structures, such as bile and pancreatic ducts. Ultimately, CLE can be used to optimize endoscopic diagnoses, thereby reducing unnecessary resections, avoiding repeated biopsies and unnecessary follow-up, and indirectly decreasing the risks and costs that are associated with repeated indiscriminative endoscopic exams. However, the interpretation of CLE images is still challenging. A standard classification system for distinguishing between normal and pathological GI conditions has only been developed for p-CLE devices; it was termed the* Miami Classification*, and it was based on a consensus that was reached among p-CLE users during a meeting that was held in Miami in 2009 [12]. Conversely, international recommendations and guidelines for other CLE systems have not yet been assessed.

The objective of the present systematic review is to summarize and analyze the current literature evaluating the sensitivity and specificity of this novel imaging modality (i.e., CLE) in detecting mucosal abnormalities. In assessing the available literature, we aimed to highlight the utility of CLE in the diagnosis of gastrointestinal and pancreatobiliary diseases, particularly in the screening or surveillance of dysplastic and neoplastic lesions, and to consider its potential future impact on daily clinical practice.

## 2. Methods

The methodological approach included the definition of search strategies, the development of selection criteria, an assessment of study quality, and the abstraction of relevant data. The PRISMA statements checklist for systematic review reporting was followed.

### 2.1. Study Inclusion Criteria

The study selection criteria were defined prior to initiating data collection to enable the proper identification of eligible studies for inclusion in the analysis.

The search was restricted to studies that were performed in humans and that were published in English. Prospective and retrospective clinical studies were both eligible for inclusion, and there were no limits based on trial duration. Review articles, case reports, commentaries, editorials, letters, and conference abstracts were not considered. Likewise, ex vivo studies were excluded. Endoscopic applications of CLE were only considered if they were being used to evaluate the following types of diseases/lesions: Barrett's esophagus; squamous cell carcinoma; esophagitis; gastroesophageal reflux disease; gastric polyps; gastric atrophy and reactive metaplasia, dysplasia, and neoplasia;* Helicobacter pylori*-related gastritis; inflammatory bowel disease (IBD); celiac disease; colonic neoplasm; colonic polyps; biliary duct disease; and benign or malignant pancreatic diseases. To be eligible for inclusion, a study must have included at least one of the following outcomes: sensitivity, specificity, positive predictive value (PPV), negative predictive value (NPV), accuracy, description of CLE indications, or mucosal features found using CLE.

### 2.2. Literature Search Strategy

A literature search was performed using the following online databases: MEDLINE (through PubMed), EMBASE, Scopus, and Cochrane Oral Health Group Specialized Register. A grey literature search was also performed by using the OpenGrey database. To increase the probability of identifying all relevant articles, a specific research equation was formulated for each database, using the following key words and/or MeSH terms: confocal laser endomicroscopy, CLE, endomicroscopy, gastrointestinal, esophagus, esophageal, bile duct, biliary, gastric, colon, colic, pancreatic, and pancreas. Additionally, reference lists from eligible studies and relevant review articles (not included in the systematic review) were cross-checked to identify additional studies.

### 2.3. Study Selection and Quality Assessment

The titles and abstracts of the retrieved studies were independently and blindly screened for relevance by two reviewers (Alessandro Fugazza and Federica Gaiani). A full article was retrieved when the information in the title and/or abstract appeared to meet the objective of this review. To enhance sensitivity, records were removed only when both reviewers excluded the record at the title screening level. All disagreements were resolved through discussions with a third and fourth reviewer (Nicola de'Angelis, Michaël Lévy). Subsequently, two reviewers (Alessandro Fugazza, Federica Gaiani) independently performed a full-text analysis and quality assessment of the selected articles. The Cochrane criteria, which are described in the Cochrane Handbook for Systematic Reviews of Interventions [17], were applied for randomized clinical trials, and the Newcastle-Ottawa Scale (NOS) [18] was used for nonrandomized studies.

### 2.4. Data Extraction and Analysis

The data that were extracted from the studies for inclusion in the systematic review were processed for qualitative and possibly quantitative analyses. The data were collected and summarized based on whether CLE was applied for gastrointestinal or pancreatobiliary diseases. Data that would enable us to perform “per patient,” “per biopsy,” and “per lesion” analyses were separately extracted whenever available. A “per patient” analysis was performed by comparing each patient's final CLE outcome against their histopathological diagnosis; similarly, “per biopsy” and “per lesion” analyses were conducted by comparing images produced by CLE with the results of histologic biopsies. All data extraction was performed by one author (Alessandro Fugazza) and was verified by a second author (Federica Gaiani), and a 100% rate of final agreement was maintained.

To perform quantitative analysis, data on sensitivity, specificity, PPV, NPV, and main findings were extracted from the eligible studies. If necessary and possible, outcome variables were calculated by the authors based on the data that were available in individually selected studies. Only on-site (i.e., real-time) data of CLE imaging analysis were considered. Meta-analyses were performed using MetaDisc (version 5.2; Clinical Biostatistics Unit, Hospital Ramon y Cajal, Madrid, Spain) [19]. Heterogeneity was assessed using *I* ^2^ statistics.

## 3. Results

**Figure 1:**
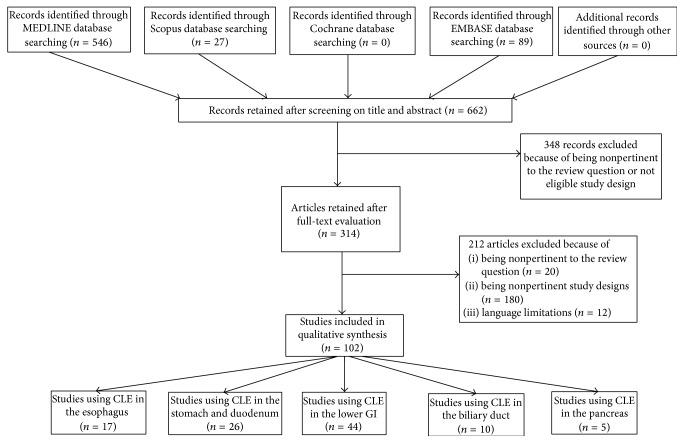
Flow chart of the electronic literature search strategy using Medline, Scopus, Embase, and other sources.

All database searches were performed without time limit until January 2015. Overall, the combined search identified 662 articles (after removing duplicates); of these, 348 were rejected based upon title and abstract evaluation. Out of the remaining articles, which underwent full-text evaluations, 212 were excluded because they either were not pertinent to the review questions, had nonrelevant study designs, or had language limitations. A final total of 102 articles were considered to be eligible for the systematic review and were evaluated by both qualitative and quantitative analyses. A flowchart of the study's identification and inclusion/exclusion processes is shown in Figure 1.

### 3.1. Study Characteristics

The studies that were included were published between 2004 and 2015. They included 8 RCTs, 85 prospective studies, and 9 retrospective studies. Twenty-eight (27.4%) studies were multicentric, with a maximum of 8 centers included. The studies were conducted in 16 different countries. The included single-center studies were conducted in Asia (*n* = 33), Europe (*n* = 31), USA (*n* = 7), and Oceania (*n* = 3); the multicentric studies were conducted in Europe + USA (*n* = 15), Europe + Asia (*n* = 2), Europe (*n* = 5), and the USA (*n* = 6).

Overall, the 102 included studies enrolled a total of 6943 patients; the number of patients per study ranged from 4 to 1572, with a median sample of 67.4 patients. The median patient age was 56.9 years (range: 0.7 to 90 years). All of the patients that underwent CLE were administered intravenous fluorescein prior to the procedure.

The outcomes produced by CLE technology are hereafter classified by organ of application, including esophagus, stomach, pancreas, biliary tree, and colon.

### 3.2. Esophagus

**Figure 2:**
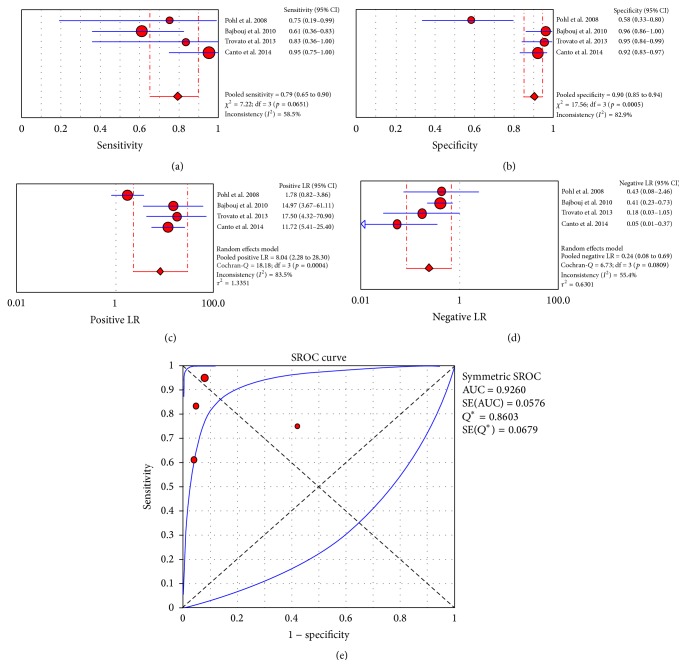
“Per patient” meta-analysis for CLE application in Barrett's esophagus: (a) pooled sensitivity, (b) pooled specificity, (c) pooled positive likelihood ratio (LR), (d) pooled negative LR, and (e) summary receiver operatic characteristic (SROC).

The most important application of CLE in the esophagus is the surveillance and evaluation of suspicious lesions in patients presenting with Barrett's esophagus (BE) [20–31]. With regard to the detection of premalignant and malignant transformations of metaplasia in BE patients, a “per biopsy” meta-analysis of 7 studies [20, 21, 23–26, 30] resulted in a pooled sensitivity of 58% (CI_95%_: 52%–63%; *I* ^2^: 95.2%), a pooled specificity of 90% (CI_95%_: 89%–91%; *I* ^2^: 96.9%), a pooled positive likelihood ratio (LR) of 11.57 (CI_95%_: 5.38–24.89; *I* ^2^: 93.7%), and a pooled negative LR of 0.23 (CI_95%_: 0.08–0.64; *I* ^2^: 98%). The area under the curve was 0.9758. A “per patient” meta-analysis based on 4 studies [21, 24, 30, 31] yielded a pooled sensitivity of 79% (CI_95%_: 65%–90%; *I* ^2^: 58.5%), a pooled specificity of 90% (CI_95%_: 85%–94%; *I* ^2^: 82.9%), a pooled positive LR of 8.04 (CI_95%_: 2.28–28.3; *I* ^2^: 83.5%), and a pooled negative LR of 0.24 (CI_95%_: 0.08–0.69; *I* ^2^: 55.4%). The area under the curve was 0.926 (Figure 2 and Table 1). Only anecdotal reports have described using CLE to detect the features of squamous cell carcinoma [32–34], reflux esophagitis [35], and nonerosive reflux disease (NERD) [36]. These latter studies were not included in the quantitative analysis (Table 1).

### 3.3. Stomach and Duodenum

**Figure 3:**
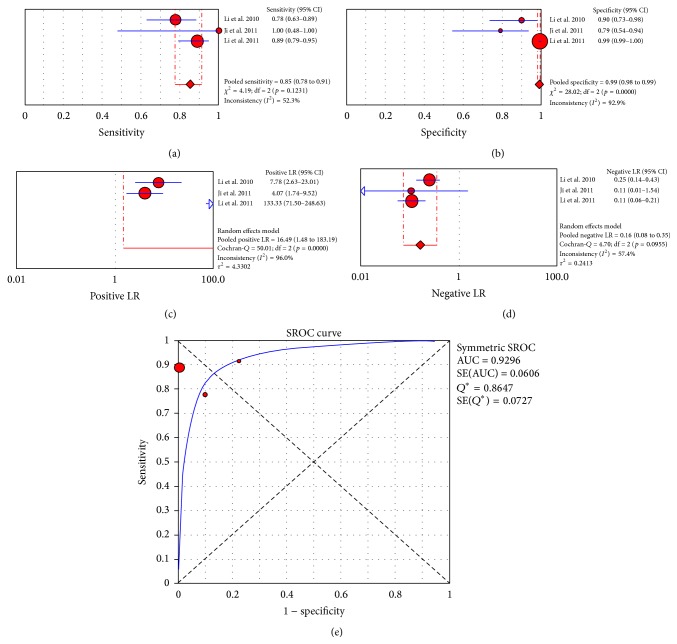
“Per patient” meta-analysis for the application of CLE in detection and diagnosis of neoplastic lesions in the stomach: (a) pooled sensitivity, (b) pooled specificity, (c) pooled positive likelihood ratio (LR), (d) pooled negative LR, and (e) summary receiver operatic characteristic (SROC) curve.

**Figure 4:**
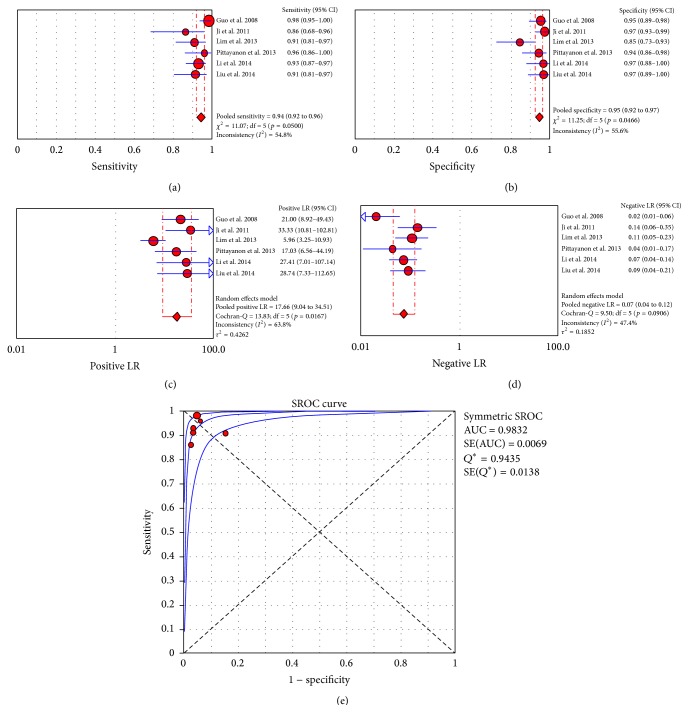
“Per biopsy” meta-analysis for the application of CLE in gastritis and gastric metaplasia: (a) pooled sensitivity, (b) pooled specificity, (c) pooled positive likelihood ratio (LR), (d) pooled negative LR, and (e) summary receiver operatic characteristic (SROC) curve.

The use of CLE in patients presenting with gastric disease has raised great interest, particularly in Asian countries, where these pathologies are highly prevalent. The primary applications of CLE with respect to the stomach and duodenum have included the detection of polyps and neoplastic lesions and the study of gastritis and metaplastic lesions [37, 41, 43, 44, 46, 47, 49, 51, 52, 54, 56]; the application of CLE has also shown utility in patients with celiac disease [60–62] and* Helicobacter pylori*-related gastritis [58] (Table 2). The performance of this innovative technique has been evaluated both alone and in comparison or in addition to other methods, such as endoscopic ultrasound (EUS) and narrow band imaging (NBI); however, the majority of these studies had descriptive aims and focused on the utility of employing CLE in targeting biopsies [40, 48]. With respect to the detection and diagnosis of polyps and neoplastic lesions, a “per patient” meta-analysis was only possible for 3 of the included studies [43, 45, 46], and it yielded a pooled sensitivity of 85% (CI_95%_: 78%–91%; *I* ^2^: 52.3%), a pooled specificity of 99% (CI_95%_: 98%-99%; *I* ^2^: 92.9%), a pooled positive LR of 16.49 (CI_95%_: 1.48–183.19; *I* ^2^: 96%), and a pooled negative LR of 0.16 (CI_95%_: 0.08–0.35; *I* ^2^: 57.4%) (Figure 3). The area under the curve was 0.929. The estimated diagnostic accuracy of CLE ranged from 85% to 98.8%. With respect to the study of gastritis and gastric metaplasia, 6 studies [50, 51, 53–56] were included in the “per biopsy” meta-analysis, which resulted in a pooled sensitivity of 94% (CI_95%_: 92%–96%; *I* ^2^: 54.8%), a pooled specificity of 95% (CI_95%_: 92%–97%; *I* ^2^: 55.6%), a positive LR of 17.66 (CI_95%_: 9.04–34.51; *I* ^2^: 63.8%), and a negative LR of 0.07 (CI_95%_: 0.04–0.12; *I* ^2^: 47.4%). The area under the curve was 0.9832 (Figure 4).

A meta-analysis of two studies regarding* Helicobacter Pylori*-related gastritis [57, 58] yielded a pooled sensitivity of 86% (CI_95%_: 76%–93%; *I* ^2^: 0%), a pooled specificity of 93% (CI_95%_: 87%–97%; *I* ^2^: 2.6%), a positive LR of 11.28 (CI_95%_: 5.4–23.57; *I* ^2^: 15.5%), and a negative LR of 0.16 (CI_95%_: 0.09–0.27; *I* ^2^: 0%) (Figure S1).

The use of CLE in assessing celiac disease, with respect to intraepithelial lymphocytes and villous atrophy, was proven to have high sensitivity and specificity. A meta-analysis performed on 3 studies [60–62] produced a pooled sensitivity of 84% (CI_95%_: 72%–92%; *I* ^2^: 71.3%), a pooled specificity of 94% (CI_95%_: 85%–99%; *I* ^2^: 66.4%), a positive LR of 9.9 (CI_95%_: 2.12–46.35; *I* ^2^: 53.9%), and a negative LR of 0.15 (CI_95%_: 0.04–0.52; *I* ^2^: 45.2%). The area under the curve was 0.9691 (Figure S2).

### 3.4. Colon

There is a wide range of applications for the use of CLE in patients with colonic diseases. These include the description of elementary and pathognomonic lesions in IBD [63–75], the detection of dysplasia/neoplasia in healed mucosa in IBD patients [76–81], and the microscopic description of colorectal polypoid lesions and neoplasms [82–102]. Although they have been less extensively studied, additional applications of CLE include obtaining microscopic descriptions of mucosa in patients with irritable bowel syndrome (IBS) [105, 106], infectious colitis (i.e., clostridium difficile infection) [104], and colitis associated with Graft versus Host Disease (GVHD) [103] (Table 3).

**Figure 5:**
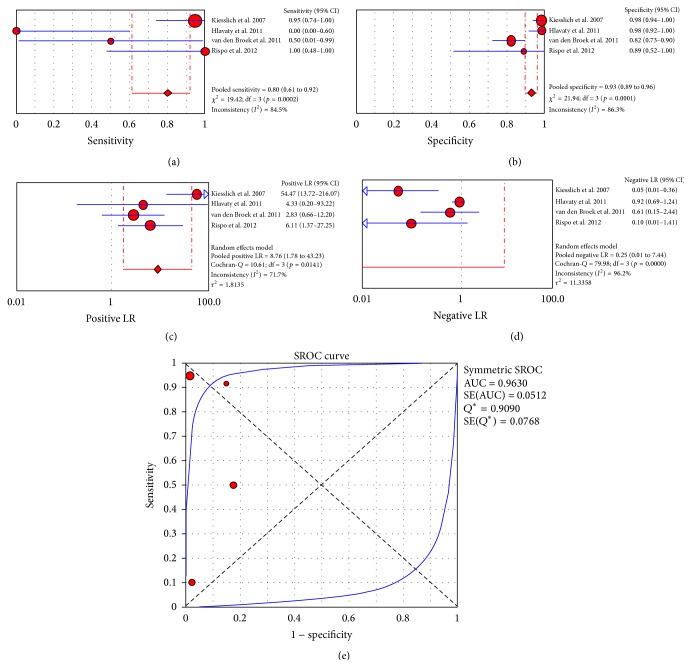
“Per lesion” meta-analysis for the application of CLE in the detection of dysplasia and neoplasia in inflammatory bowel disease patients: (a) pooled sensitivity, (b) pooled specificity, (c) pooled positive likelihood ratio (LR), (d) pooled negative LR, and (e) summary receiver operatic characteristic (SROC) curve.

**Figure 6:**
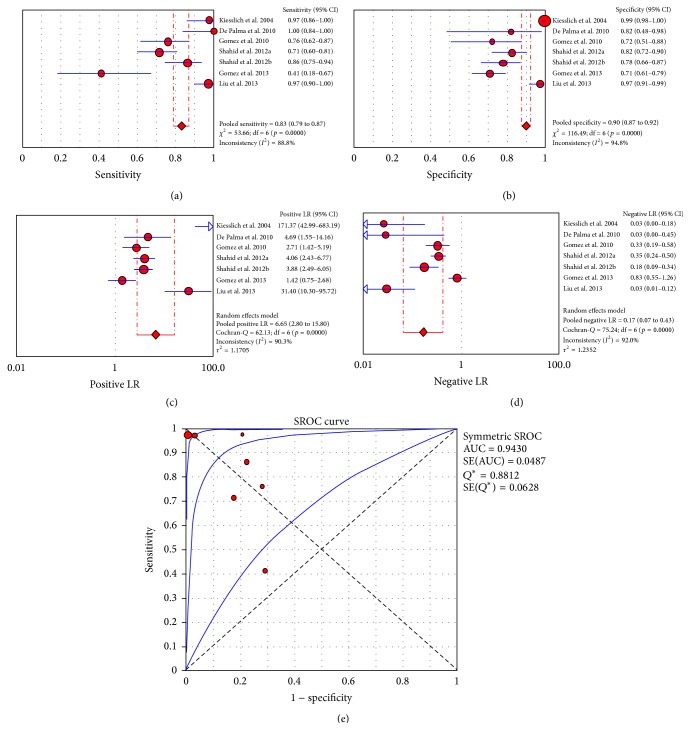
“Per lesion” meta-analysis for the application of CLE in colorectal neoplasms and malignant foci in polypoid lesions: (a) pooled sensitivity, (b) pooled specificity, (c) pooled positive likelihood ratio (LR), (d) pooled negative LR, and (e) summary receiver operatic characteristic (SROC) curve.

A meta-analysis of 4 studies [77, 79–81] that investigated dysplasia and neoplasia in IBD patients produced a pooled “per lesion” sensitivity of 80% (CI_95%_: 61%–92%; *I* ^2^: 84.5%), a pooled specificity of 93% (CI_95%_: 89%–96%; *I* ^2^: 86.3%), a pooled positive LR of 8.76 (CI_95%_: 1.78–44.23; *I* ^2^: 71.7%), and a pooled negative LR of 0.25 (CI_95%_: 0.01–7.44; *I* ^2^: 96.2%). The area under the curve was 0.9630 (Figure 5). A meta-analysis of 7 studies that investigated colorectal neoplasms and polyps produced a pooled “per lesion” sensitivity of 83% (CI_95%_: 79%–87%; *I* ^2^: 88.8%), a pooled specificity of 90% (CI_95%_: 87%–92%; *I* ^2^: 94.8%), a pooled positive LR of 6.65 (CI_95%_: 2.8–15.8; *I* ^2^: 90.3%), and a pooled negative LR of 0.17 (CI_95%_: 0.07–0.43; *I* ^2^: 92%). The area under the curve was 0.9430 (Figure 6).

CLE has also been widely applied to describe IBD morphologic features, although it was not possible to perform a quantitative analysis of this application. In this particular indication, CLE appears to be a safe and feasible diagnostic tool for imaging bowel morphology, assessing disease activity, and predicting therapeutic responses.

### 3.5. Biliary Duct

In biliary tract and pancreatic cysts, routine forceps are not accurate for sampling and pathology exam fails to address diagnosis. CLE with in situ diagnosis might be a valuable alternative. However, few studies have been carefully conducted.

**Figure 7:**
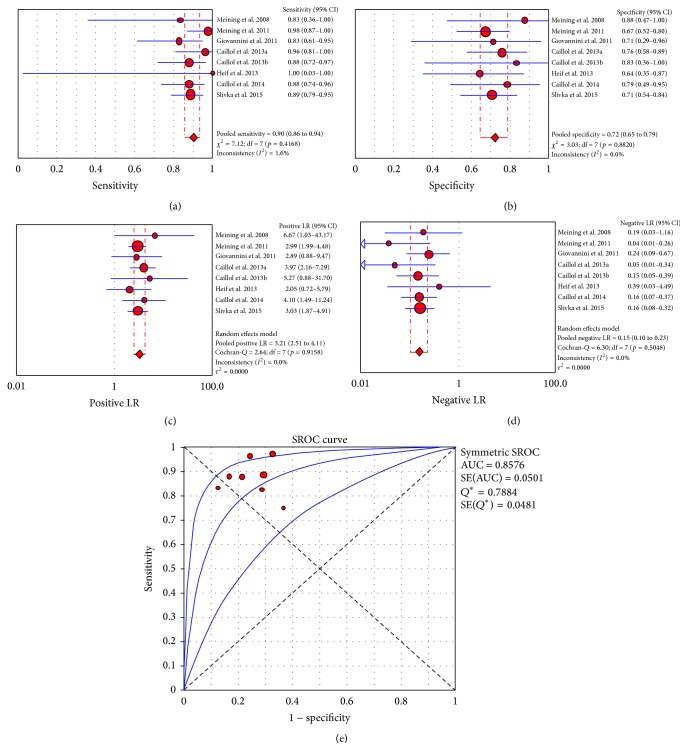
“Per patient” meta-analysis for biliary duct application of CLE: (a) pooled sensitivity, (b) pooled specificity, (c) pooled positive likelihood ratio (LR), (d) pooled negative LR, and (e) summary receiver operatic characteristic (SROC) curve.

CLE has been applied for the diagnosis of common biliary duct lesions and to distinguish between benign and malignant strictures in patients with indeterminate biliary stenosis [2, 107–115] (Table 4). These applications were prospectively evaluated in several studies; of these, we performed a meta-analysis of 8 studies [107–109, 111–115], which resulted in a pooled sensitivity of 90% (CI_95%_: 86%–94%; *I* ^2^: 1.6%), a pooled specificity of 72% (CI_95%_: 65%–79%; *I* ^2^: 0%), a pooled positive LR of 3.21 (CI_95%_: 2.55–4.11; *I* ^2^: 0%), and a pooled negative LR of 0.15 (CI_95%_: 0.10–0.23; *I* ^2^: 0%). The area under the curve was 0.8578 (Figure 7).

### 3.6. Pancreas

Currently, in humans, the use of nCLE is exclusively applied to pancreatic tissue. The literature concerning this technique includes a small number of studies that examined the characterization of pancreatic lesions, such as pancreatic cystic neoplasms [14, 15, 117], indeterminate pancreatic duct strictures [116], and serous cystadenomas [118] (Table 5). A meta-analysis of two studies [14, 117] that applied nCLE for the diagnosis of pancreatic cyst neoplasms produced a pooled sensitivity of 68% (CI_95%_: 55%–80%; *I* ^2^: 79.8%), a pooled specificity of 90% (CI_95%_: 74%–98%; *I* ^2^: 82.4%), a pooled positive LR of 6.72 (CI_95%_: 0.94–47.89; *I* ^2^: 52%), and a pooled negative LR of 0.30 (CI_95%_: 0.10–0.84; *I* ^2^: 60.6%) (Figure S3).

### 3.7. Study Quality Assessment

Based on the GRADE system, the overall quality of the evidence included in our analysis was judged as low (10 studies had a very low level of quality; 59 were low; 31 were moderate; and 2 were high). The risk of bias in the comparative randomized and nonrandomized studies was evaluated by two independent reviewers (Alessandro Fugazza, Federica Gaiani) based on Cochrane and NOS criteria. Specifically, two RCTs [55, 77] were classified as having a low risk of bias, and 6 RCTs had a high risk of bias [22, 25, 27, 28, 31, 54] (Table S2). Based on NOS criteria, 5 studies [29, 52, 62, 70, 74] were classified as having a low risk of bias, and 17 [36, 39, 48, 59–61, 63, 66–69, 71, 72, 78, 94, 106, 112] had a high risk of bias (Table S3).

## 4. Discussion

To the best of our knowledge, this is the first systematic review to analyze the diagnostic accuracy of CLE across all of the applications for which it has been used.

A crucial difference between CLE and alternative endoscopic techniques is that CLE does not merely identify a lesion, but it also enables the discrimination of benign or malignant features via direct and immediate microscopic investigation, which can be performed simultaneously with endoscopic examination [119]. Despite this, CLE is still viewed as an expensive tool that requires standardized criteria for the diagnosis of select pathologies and needs specific training to interpret CLE images before it can be implemented as a routine diagnostic tool.

### 4.1. CLE in the Esophagus

Several studies have validated the diagnostic and therapeutic role of CLE with respect to premalignant lesions and cancers of the upper GI tract. In a feasibility study, the use of pCLE demonstrated an up to 92% accuracy rate in detecting malignant and premalignant modifications of GI mucosa compared to conventional histopathology [120]. However, the primary application of CLE in esophageal tissue has been for the detection of high-grade dysplasia and cost-effective treatment strategies of BE. In particular, CLE has been used for follow-up of BE patients presenting with HG dysplasia and to define the lateral extent of neoplasias prior to therapy.

It has been demonstrated that when using endomicroscopy for the surveillance of BE, the number of required biopsies can be significantly decreased by up to 87% [22], as this technique produces a higher diagnostic yield for the detection of neoplasia compared to random biopsy. This improved yield directly results from the need to sample only the suspicious areas that have been identified by CLE [22, 31]. However, what the optimal surveillance biopsy procedure is in BE patients remains unclear. Current surveillance programs, such as the four-quadrant Seattle biopsy protocol, are relatively expensive and time-consuming [29]. The Preservation and Incorporation of Valuable Endoscopic Innovation (PIVI) initiative that was recently implemented by the American Society of Gastrointestinal Endoscopy (ASGE) [121] recommends that, before replacing the current Seattle protocol, a targeted imaging technique should have a per patient sensitivity of at least 90%, an NPV of at least 98%, and a specificity of at least 80% in the detection of high-grade dysplasia or early adenocarcinoma. The present meta-analysis demonstrated that CLE yields a “per biopsy” pooled sensitivity of 58% and a pooled specificity of 90%, which was slightly increased to 79% sensitivity in the “per patient” analysis. Therefore, based on the PIVI initiative requirements, using CLE for the surveillance of BE does not appear to be sensitive enough to replace the Seattle biopsy protocol. However, a recent multicenter RCT showed that combining CLE with high-definition WLE surpassed the PIVI threshold, with a per patient sensitivity of 95%, an NPV of 98% and a specificity of 92% [31, 122]. Thus, the combined use of CLE with high-definition WLE or NBI may be considered a valuable diagnostic tool for premalignant and malignant lesions. Nevertheless, prospective medicoeconomic studies have yet to be conducted.

### 4.2. CLE in the Stomach and Duodenum

CLE has been used for the description and detection of several gastric diseases, including polyps, metaplasia, and neoplastic lesions, as well as for the surveillance of gastric resections,* Helicobacter pylori*-related gastritis, and celiac disease. However, knowledge surrounding the application of CLE to the stomach remains limited (although promising), and further clinical trials are needed to support the clinical impact of employing CLE in the diagnosis and management of total gastric atrophy.

The usefulness of CLE is especially evident with regard to targeting biopsies of specific pathological mucosal areas. Indeed, a recent prospective study comparing NBI, chromoendoscopy (CE), and CLE for the diagnosis of atrophic gastritis found NBI to be equivalent to CE in classifying gastric pits, whereas CLE had higher sensitivity, specificity, and accuracy than CE [53]. Furthermore, with respect to the detection of gastric intestinal metaplasia (GIM), CLE demonstrated high diagnostic yields and a substantial superiority over conventional endoscopy; with CLE, the number of biopsies needed to confirm GIM was about one-third of what was needed compared to using WLE and standard biopsies [50, 55] also suggesting a gain of time. This may be also the case in those patients at very high risk of early carcinoma (Lynch syndrome, CDH mutated individuals).

CLE has also been used for descriptive purposes; a blinded prospective study investigating gastric pit patterns provided an obvious distinction between normal mucosa, chronic inflammation, atrophy, and neoplastic mucosa and demonstrated a sensitivity and specificity for predicting gastric atrophy of 83.6% and 99.6%, respectively, whereas the corresponding values for predicting gastric cancer were 90.0% and 99.4% [123].

With respect to gastric polyps and neoplastic lesions, CLE has demonstrated several promising applications, including distinguishing between adenomas and hyperplastic polyps [42], the identification of microvascular patterns [39], follow-up after resection [45], and the diagnosis of cancer at different stages. The majority of studies have indicated that CLE has high diagnostic yields, and elevated interobserver agreement rates were evident in most cases [42]. However, CLE accuracy can be limited by the acquisition of good quality images [38], which may not be always possible. The present meta-analysis demonstrated that CLE yields remarkable pooled sensitivity and specificity, reaching values of 85% and 99%, respectively, in a “per patient” analysis.

Interestingly, CLE has also been applied to assess duodenal histology in patients with celiac disease. Employing CLE offers the prospect of diagnosing celiac disease during ongoing endoscopy and enables targeting biopsies to be performed in abnormal mucosa, thereby increasing diagnostic yield. However, although CLE appears to be sensitive and specific at detecting increased numbers of intraepithelial lymphocytes and villous atrophy, the evidence that CLE is effective for such applications remains scarce. More likely, CLE methodology would need to be improved before it could be routinely used in patients with celiac disease.

Overall, applying CLE to examine the stomach and duodenum demonstrated high sensitivity, specificity, accuracy, and positive and negative predictive values in comparison with both histopathology and other endoscopic techniques (e.g., WLE, NBI, and CE). However, these data are based on a limited number of publications and therefore caution should be used when interpreting their results.

### 4.3. CLE in the Colon

The increasing incidence of colorectal cancers in parallel with the increased use of colonoscopy screening necessitates continued improvements in diagnostic accuracy and precocity. Therefore, the advent of CLE has been considered to be an important and valuable innovation for the management of colorectal neoplasms and inflammatory diseases [77].

Based on the present meta-analysis, the use of CLE in the detection of colorectal neoplasms and malignant foci in polypoid lesions is associated with a pooled sensitivity of 83% and a pooled specificity of 90%. This confirms the robust diagnostic power of CLE that has been observed in previous studies. Moreover, when compared to NBI, pCLE exhibited higher sensitivity (86% versus 64%) and similar overall accuracy (82% versus 79%), although it also exhibited lower specificity (78% versus 92%) [96]. Interestingly, the diagnostic accuracy of CLE does not appear to be influenced by operator expertise in the evaluation of confocal images; in contrast, learning how to perform CLE appears to involve only a short learning curve [102]. In addition to its role as a diagnostic tool, CLE is useful for evaluating the presence of residual tumor left behind following endoscopic treatment of colon polyps and does not require waiting for biopsy results. If there is an eventual need for reintervention via a complementary resection, it can therefore be carried out during a single endoscopic session [97].

CLE also demonstrated high applicability and superiority over standard endoscopy in the study of IBD. In particular, CLE can be used in the assessment of disease activity [70], in the prediction of relapse [71, 74], and in the description of mucosal alterations such as epithelial gaps, all of which are useful toward enhancing the comprehension of new pathogenic features that develop in patients with IBD [66]. However, the detection of neoplastic transformations in the background of chronic inflammation in IBD patients remains highly challenging. Conversely, using CLE in patients with IBD is promising approach, as it offers the possibility of directly observing microvessels by immunostaining and therefore may serve as a foundation for the development targeted antiangiogenic therapies [91, 101].

Applying CLE to patients GVHD, infectious colitis and irritable bowel syndrome has been less extensively studied; however, this technique has demonstrated good performance in these indications (100% specificity and less invasiveness in comparison with standard diagnosis technique), although its standards remain outside of current guidelines [103, 106].

### 4.4. CLE in the Biliary Duct

With respect to the biliary duct, the addition of CLE to histological examination results in a significant increase in diagnostic reliability [114]. Malignant pancreatobiliary strictures are difficult to diagnose, and up to 30% of patients with cholangiocarcinoma have negative sampling [111]. This is because, unlike other tumors, the majority of cholangiocarcinomas grow along bile duct walls rather than radially forming a mass [124]. Currently, biliary strictures are staged using a combination of endoscopic ultrasound and advanced imaging techniques, such as computed tomography (CT), magnetic resonance imaging (MRI), or EUS, whereas endoscopic retrograde cholangiopancreatography (ERCP) is typically used for tissue sampling, including biopsy and cytological brushing. However, the current sensitivity of each of these methods is quite low, ranging from 20% to 60% [125–127].

The present meta-analysis demonstrated that combining CLE with ERCP yields high sensitivity (90%) in the assessment of biliary strictures. This supports the ASGE guidelines and demonstrates that CLE is a useful tool for differentiating benign from malignant biliary strictures in patients with biliary neoplasia [128].

### 4.5. CLE in the Pancreas

The accurate diagnosis of pancreatic cystic neoplasms continues to be problematic despite technological improvements [117]. The application of an optical needle biopsy by using nCLE may significantly improve the discrimination of mucinous and nonmucinous cysts. Based on 2 studies that were included in the meta-analysis, CLE yields a pooled sensitivity of 68% and pooled specificity of 90%, which might be suboptimal and influenced by the high heterogeneity that was observed between the studies. However, the current standard diagnostic techniques, such as imaging, EUS, fluid analysis (e.g., chemistry, tumor markers), and cytology [14, 118], which when combined lead to a correct diagnosis in not more than 79% of cases, are also far from being satisfactory diagnostic procedures [129].

It should be noted that the literature regarding the use of nCLE for the diagnosis of pancreatic cystic neoplasms is scarce [14, 117] and is mainly focused on serous cystadenoma [118]. Further studies are needed to improve nCLE diagnostic power and accuracy.

### 4.6. Study Limitations and Future Directions

The present systematic review and meta-analysis attempted to summarize the current literature on the applications of CLE in gastrointestinal and pancreatobiliary diseases. Although a considerable number of studies were retrieved overall, the total evidence per organ is rather scarce and is often too low to draw definitive conclusions. Moreover, high heterogeneity was observed in many of our pooled data analyses, which indicates that caution is required when interpreting their results. Finally, the included studies were primarily conducted in specialized centers; thus, CLE outcomes cannot be generalized to tertiary care centers or nonspecialized institutions.

Despite these limitations, the present systematic review and meta-analysis highlights the novel and unique advantages of using CLE to provide real-time histological examination during diagnostic and therapeutic procedures. Future clinical trials should aim to improve the diagnostic accuracy of CLE in all of its possible applications, to institutionalize training programs to standardize the interpretation of CLE images, and to reduce procedure related costs and limitations to increase its application. By improving and implementing CLE techniques during routine clinical practice, we believe that in the near future CLE not only will become part of the diagnostic arsenal of the gastroenterologist/endoscopist but also may find application in related fields such as minimally invasive surgical techniques.

## 5. Conclusions

In gastrointestinal and pancreatobiliary diseases, endoscopy-associated new technologies should offer the possibility to make clear diagnosis when routine procedures make it difficultly be cost-effective with clear impact on the choice of endoscopy versus surgical therapies for macroscopic lesions and achieve early detection of malignancies in those individuals with very high risk of cancer development. CLE is one of these new technologies able to address the challenge. The overall sensitivity, specificity, accuracy, and predictive values of CLE are favorable and were often found to be superior in comparison with standard endoscopy plus histopathology. However, the widespread use of CLE remains limited by its low availability, high costs, and need for trained personnel. Moreover, there is a need for further clinical trials, including medicoeconomic evaluations, to assess the applicability and implementation of CLE in routine clinical practice, as currently very few such studies exist.

## Supplementary Material

The Supplementary Material contains:
Figures S1,S2,S3 describing the meta-analysis of studies about H. pylori infection, Celiac disease and pancreatic cyst neoplasms.
Table S1 describes the characteristics of different CLE devices.
Tables S2, S3 describe the Quality Assessment of the all the studies included in the review based on the Cochrane criteria for randomized clinical trials and the Newcastle-Ottawa Scale (NOS) for nonrandomized studies.

## Figures and Tables

**Table 1:** Summary of the studies applying CLE for esophageal lesion screening and diagnosis (“per biopsy” or “per patient” analysis).

Author and year	Country	*N*	Mean age or range	Sensitivity (%)	Specificity (%)	PPV (%)	NPV (%)	Accuracy (%)	Main findings

Barrett's esophagus									
Kiesslich et al. 2006 [20]	Germany	63 patients 156 biopsies	58.7	92.9 n/a	98.4 n/a	92.9 n/a	98.4 n/a	97.4 n/a	CLE may be helpful in the management of patients with BE because gastric, Barrett's epithelium, and Barrett's associated neoplastic changes can be diagnosed with high accuracy.
Pohl et al. 2008 [21]	Germany	38 patients 201 biopsies	62.1	75 80	57.9 94.1	n/a 44.4	91.7 98.8	60.9 93.3	CLE showed a high NPV for the diagnosis of endoscopically invisible neoplasia in BE.
Dunbar et al. 2009 [22]	USA	39 patients	64	n/a	n/a	n/a	n/a	n/a	CLE increases diagnostic yield for neoplasia and reduces the number of mucosal biopsies.
Wallace et al. 2010 [23]	USA Germany France	5 patients 20 biopsies	38–86	n/a 88	n/a 96	n/a n/a	n/a n/a	n/a 92	pCLE has very high accuracy and reliability for the diagnosis of neoplasia in BE.
Bajbouj et al. 2010 [24]	Germany	68 patients 703 biopsies	60	60 12	95 95	67 18	93 92	n/a n/a	pCLE can be regarded as noninferior to endoscopic biopsy but, for its low PPV and sensitivity, may currently not replace standard biopsy techniques for the diagnosis of BE and associated neoplasia.
Sharma et al. 2011 [25]	USA France Germany	101 patients 874 biopsies	65.1	n/a 62.5	n/a 92.7	n/a 57.7	n/a 94.0	n/a n/a	pCLE combined with HD-WLE significantly improved the ability to detect neoplasia in BE patients compared with HD-WLE.
Jayasekera et al. 2012 [26]	Australia	50 patients 1117 biopsies	66	n/a 75.7* *	n/a 80.0* *	n/a 98.1	n/a 19.4	n/a 79.9* *	Minimal additional diagnostic impact of CLE above HD-WLE and NBI in the assessment of BE.
Wallace et al. 2012 [27]	USA France UK	164 patients	67.9 69.6	n/a	n/a	n/a	n/a	n/a	The addition of pCLE to high-definition white light imaging does not improve diagnostic accuracy nor clinical outcomes in patients undergoing ablation or resection for BE.
Nguyen et al. 2012 [28]	USA	18 patients	72.6	n/a	n/a	n/a	n/a	n/a	Endoscopists with minimal experience in CLE can effectively use this technology for targeted biopsy, decreasing the need for intense tissue sampling without lowering the diagnostic yield in detecting dysplasia.
Bertani et al. 2013 [29]	Italy	100 patients	59.7	100	83	67	100	n/a	Incident dysplasia can be more frequently detected by pCLE than by HD-WLE in BE. The higher dysplasia detection rate provided by pCLE could improve the efficacy of BE surveillance programs.
Trovato et al. 2013 [30]	Italy	48 patients 422 biopsies	54	83.3 93.3	95.2 98.2	71.4 66.6	97.6 99.7	93.7 98.1	CLE can provide in vivo diagnosis of Barrett tumor-associated esophagus leading to significant improvements in screening and monitoring of in vivo BE.
Canto et al. 2014 [31]	USA Germany	192 patients 978 biopsies	62	95 86	92 93	77 65	98 98	93 92	Real-time CLE and TB after HD-WLE can improve the diagnostic yield and accuracy for neoplasia and significantly impact in vivo decision-making by altering the diagnosis and guiding therapy.

Squamous cell carcinoma									
Pech et al. 2008 [32]	Germany	21 patients	64	100	87.5	93	100	95	CLE is able to provide virtual histology of early squamous cell cancers with a high degree of accuracy and can facilitate rapid diagnosis during routine endoscopy.
Liu et al. 2009 [33]	China	27 patients	61.1	n/a	n/a	n/a	n/a	n/a	CLE can be used to distinguish cancerous from normal epithelium, which gives it potential value for early detection of esophageal carcinoma.
Iguchi et al. 2009 [34]	Japan	15 patients	65.9	n/a	n/a	n/a	n/a	n/a	Scoring and quantification of CLE images may be useful for the differential diagnosis and determination of superficial invasion by squamous cell carcinoma.

Reflux esophagitis, nonerosive reflux disease (NERD)									
Venkatesh et al. 2012 [35]	Australia	23 patients	7.6 12	n/a	n/a	n/a	n/a	n/a	Measurement of the S-P distance by CLE will enable real-time diagnosis of GERD-related esophagitis during ongoing endoscopy.
Chu et al. 2012 [36]	China	46 patients	48.12 45.23	n/a	n/a	n/a	n/a	n/a	CLE represents a useful and potentially significant improvement over standard endoscopy to examine the microalterations of the esophagus in vivo.

**Table 2:** Summary of the studies applying CLE for gastric and duodenal lesion screening and diagnosis (“per biopsy” analysis or “per patient” analysis).

Author and year	Country	*N*	Mean age or range	Sensitivity (%)	Specificity (%)	PPV (%)	NPV (%)	Accuracy (%)	Main findings
Polyps and neoplastic lesions of the stomach									
Kakeji et al. 2006 [37]	Japan	9 patients	n/a	n/a	n/a	n/a	n/a	n/a	CLE could be a new screening tool for early detection of malignancy.
Kitabatake et al. 2006 [38]	Japan	27 patients	70	81.8	97.6	n/a	n/a	97.4	CLE may allow virtual biopsy in the future with more stable imaging condition.
Liu et al. 2008 [39]	China	24 patients	n/a	n/a	n/a	n/a	n/a	n/a	CLE observations of vascular architecture may be assistant in the identification of early gastroesophageal cancer.
Gheorghe et al. 2009 [40]	Romania	11 patients	59.7	n/a	n/a	n/a	n/a	n/a	CLE and endoscopic ultrasound are successfully associated for histological assessment, disease staging, and optimal therapeutic decision.
Banno et al. 2010 [41]	Japan	29 patients	68.6	86.4	83.3	n/a	n/a	85	The CLE diagnosis of the mucin phenotype in gastric cancers was limited to intestinal and mixed phenotypes but may be useful for the diagnosis of mucin phenotype and differential diagnosis.
Li et al. 2010 [42]	China	66 patients	17–81	n/a	n/a	n/a	n/a	90	CLE demonstrates high accuracy of differentiating hyperplastic polyps and adenomas.
Li et al. 2010 [43]	China	108 patients	57	77.8	81.8	n/a	n/a	n/a	CLE is an acceptable and potentially useful technology for the identification and grading of gastric intraepithelial neoplasia in vivo. The diagnostic accuracy needs to be improved.
Jeon et al. 2011 [44]	Korea	31 patients	61.6	n/a	n/a	n/a	n/a	94.2	CLE demonstrates a high diagnostic accuracy for gastric epithelial neoplasia. The use of CLE could possibly reduce the number of unnecessary biopsies and mistaken diagnoses before endoscopic submucosal dissection.
Ji et al. 2011 [45]	China	24 patients	61.2	100	89.5	n/a	n/a	91.7	CEL has high accuracy for prediction of remnant tissue after endoscopic mucosal resection and may lead to significant improvements in clinical surveillance after endoscopic resection.
Li et al. 2011 [46]	China	1572 patients	58.1	88.9	99.3	85.3	99.5	98.8	CLE can be used to identify gastric superficial cancer/HGIN lesions with high validity and reliability.
Lim et al. 2011 [47]	China	36 patients	>50	95.2	93.3	n/a	n/a	n/a	Experience in CLE was associated with greater accuracy in the diagnosis of gastric intestinal metaplasia.
Wang et al. 2012 [48]	China	59 patients	63.1	90.6	84.6	n/a	n/a	88	CLE demonstrates high diagnostic accuracy, sensitivity, and specificity for diagnostic classification of gastric intraepithelial neoplasia.
Bok et al. 2013 [49]	Korea	46 patients	65.3	n/a	n/a	n/a	n/a	91.7	pCLE can provide an accurate diagnosis for superficial gastric neoplasia, and it has the potential to compensate for the inherent limitations of a conventional endoscopic biopsy.

Gastritis and gastric metaplasia									
Guo et al. 2008 [50]	China	53 patients 267 biopsies	51.2	n/a 98.1	n/a 95.3	n/a 96.9	n/a 97.14	n/a n/a	CLE is a useful and potentially important method for the diagnosis and classification of gastric intestinal metaplasia in vivo.
Ji et al. 2011 [51]	China	76 patients 145 biopsies	48.8	n/a 86.2	n/a 97.4	n/a 89.3	n/a 96.6	n/a 92.8	CLE is useful for identifying gastric metaplasia. These findings could have potential applicability for duodenal screening and suggest a possible targeting therapy in functional dyspepsia.
Ji et al. 2012 [52]	China	42 patients	50.4	n/a	n/a	n/a	n/a	n/a	CLE allows functional imaging of mucosal barrier defects. Gastric intestinal metaplasia is associated with an impaired paracellular barrier irrespective of *H. pylori* eradication.
Lim et al. 2013 [53]	China	20 patients 125 biopsies	62.5	n/a 90.9	n/a 84.7	n/a n/a	n/a n/a	n/a 88	pCLE was superior to autofluorescence imaging and white-light endoscopy for diagnosing gastric intestinal metaplasia. Off-site review improved performance of pCLE.
Pittayanon et al. 2013 [54]	Thailand	60 patients 120 biopsies	62.8	n/a 96	n/a 90	n/a 86	n/a 97	n/a 92	Combining pCLE with magnifying flexible spectral imaging color enhancement (ME-FICE) provides high sensitivity and NPV for gastric intestinal metaplasia detection. The prompt histology reading by this technique may avoid unnecessary biopsy.
Li et al. 2014 [55]	China	168 patients 492 biopsies	55	n/a 91.6	n/a 96.7	n/a n/a	n/a n/a	n/a n/a	CLE with targeted biopsies is superior to white-light endoscopy with standard biopsies for the detection and surveillance of gastric intestinal metaplasia. The number of biopsies needed to confirm gastric intestinal metaplasia is about one-third of that needed with white-light endoscopy with standard biopsies.
Liu et al. 2015 [56]	China	87 patients 130 biopsies	49.7	n/a 91.9	n/a 96.8	n/a 90.4	n/a 97.3	n/a 95.6	CLE is reliable for real-time assessment of atrophic gastritis and is also able to differentiate metaplastic from nonmetaplastic atrophy.

Helicobacter pylori related gastritis									
Ji et al. 2010 [57]	China	103 patients	48.4	89.2	95.7	94.3	91.7	92.8	CLE during endoscopy can accurately identify specific cellular and subcellular changes of the surface gastric mucosa induced by *H. pylori* infection.
Wang et al. 2010 [58]	China	118 patients	49.8	82.9	90.9	n/a	n/a	n/a	CLE can accurately show the histological severity of *H. pylori* infection-associated gastritis.

Duodenal inflammatory bowel disease									
Lim et al. 2014 [59]	Germany Japan UK	25 patients	n/a	n/a	n/a	n/a	n/a	n/a	CLE can detect epithelial damage and barrier loss in the duodenum of Crohn's Disease and Ulcerative Colitis patients that is not apparent on conventional endoscopy or histology.

Celiac disease									
Leong et al. 2008 [60]	Australia	31 patients	41	94	92	n/a	n/a	n/a	CLE can effectively diagnose and evaluate celiac disease severity in vivo with potential to improve endoscopy efficiency.
Günther et al. 2010 [61]	Germany	60 patients	57	73	100	n/a	n/a	n/a	The assessment of duodenal histology by CLE in patients with celiac disease is sensitive and specific in determining increased numbers of intraepithelial lymphocytes and villous atrophy but insufficient in respect of crypt hyperplasia. For routine use of CLE in patients with celiac disease, the technique would need to be improved.
Venkatesh et al. 2010 [62]	UK	19 patients	8.35	100	80	81	n/a	n/a	CEL offers the prospect of diagnosis of celiac disease during ongoing endoscopy. It also enables targeting biopsies to abnormal mucosa and thereby increasing the diagnostic yield, especially when villous atrophy is patchy in the duodenum.

*N* stands for the number of patients enrolled in the study; n/a, not available or not applicable; pCLE, probe-based confocal laser endomicroscopy; CLE, confocal laser endomicroscopy; PPV, positive predictive value; NPV, negative predictive value; and BE, Barrett's esophagus.

**Table 3:** Summary of the studies evaluating CLE in lower gastrointestinal disease screening and diagnosis (“per patient” and “per lesion” analysis).

Author and year	Country	*N*	Mean age or range	Sensitivity (%)	Specificity (%)	PPV (%)	NPV (%)	Accuracy (%)	Main findings
Inflammatory bowel disease									
Watanabe et al. 2008 [63]	Japan	31 patients	n/a	n/a	n/a	n/a	n/a	n/a	Images obtained with CLE provide equivalent information to conventional histology.
Trovato et al. 2009 [64]	Italy	18 patients 253 lesions	70	n/a 94.1	n/a 100	n/a n/a	n/a n/a	n/a 94.4	Endomicroscopy may be helpful in the evaluation of morphologic changes in ileal pouch.
Li et al. 2010 [65]	China	73 patients	50.4	n/a	n/a	n/a	n/a	n/a	CLE is reliable for real-time assessment of inflammation activity in UC by evaluation of crypt architecture, microvascular alterations, and fluorescein leakage.
Liu et al. 2011 [66]	Canada USA	57 patients	46.6	n/a	n/a	n/a	n/a	n/a	Epithelial gap density visualized by CLE is significantly increased in patients with IBD compared with controls.
Moussata et al. 2011 [67]	France, Germany, and UK	21 patients 26 lesions	n/a	n/a 89	n/a 100	n/a 100	n/a 80	n/a 92.3	CLE is a new tool that can image intramucosal bacteria in vivo in patients with IBD.
Kiesslich et al. 2012 [68]	Germany, France, UK, and China	58 patients	n/a	62.5	91.2	n/a	n/a	79	Cell shedding and barrier loss detected by CLE predict relapse of IBD and have potential as a diagnostic tool for the management of the disease.
Krauss et al. 2012 [69]	Germany	146 patients	34.9	n/a	n/a	n/a	n/a	n/a	CLE allows the analysis of the subsurface structure of lymphoid follicles, those surrounded by a red ring may represent an early marker of CD.
Neumann et al. 2012 [70]	Germany	72 patients	39	n/a	n/a	n/a	n/a	n/a	CLE has the potential to significantly improve diagnosis of CD compared with standard endoscopy.
Turcotte et al. 2012 [71]	Canada, USA	41 patients	41.1	n/a	n/a	n/a	n/a	n/a	Increased epithelial gaps in the small intestine as determined by pCLE are a predictor for future hospitalization or surgery in IBD patients.
Musquer et al. 2013 [72]	France, USA	16 patients	35.5	n/a	n/a	n/a	n/a	n/a	CLE allows quantitative analysis of colonic pit structure in healthy and CD patients.
Atreya et al. 2014 [73]	Germany	25 patients	41.6	n/a	n/a	n/a	n/a	n/a	Molecular imaging with fluorescent antibodies has the potential to predict therapeutic responses to biological treatment in CD and autoimmune or inflammatory disorders.
Buda et al. 2014 [74]	Italy, Germany, and UK	38 patients	52	n/a	n/a	n/a	n/a	n/a	In vivo intramucosal changes detected by CLE in UC remittent patients can predict disease relapse.
Li et al. 2014 [75]	China	43 patients	44	95.7	85	n/a	n/a	90.7	CLE is comparable to conventional histology in predicting relapse in patients with UC.

Dysplasia/neoplasia in inflammatory bowel disease									
Hurlstone et al. 2007 [76]	UK	36 patients	56	n/a	n/a	n/a	n/a	97	Adenoma Like Masses and Displasia Associated Lesional Masses can be differentiated by CLE with a high overall accuracy in patients with Ulcerative Colitis.
Kiesslich et al. 2007 [77]	Germany	153 patients 134 lesions	44	n/a 94.7	n/a 98.3	n/a 90	n/a 99.1	n/a 97.8	Chromoscopy-guided endomicroscopy can determine if Ulcerative Colitis should undergo biopsy examination, increasing the diagnostic yield and reducing the need for biopsy examinations.
Günther et al. 2011 [78]	Germany	150 patients	48.3	n/a	n/a	n/a	n/a	n/a	CLE targeted biopsies led to higher detection rates of intraepithelial neoplasia in patients with long-standing UC.
Hlavaty et al. 2011 [79]	Slovakia	30 patients 68 lesions	n/a	n/a 100	n/a 98.4	n/a 66.7	n/a 100	n/a n/a	CLE targeted biopsies are superior to random biopsies in the screening of intraepithelial neoplasia in patients with inflammatory bowel disease.
van den Broek et al. 2011 [80]	Netherlands	22 patients 87 lesions	54	n/a 65	n/a 82	n/a n/a	n/a n/a	n/a 81	pCLE for UC surveillance is feasible with reasonable diagnostic accuracy.
Rispo et al. 2012 [81]	Italy	51 patients 14 biopsies	52	n/a 100	n/a 90	n/a 83	n/a 100	n/a n/a	CLE is an accurate tool for the detection of dysplasia in long-standing Ulcerative Colitis, limiting the need of biopsies.

Colorectal neoplasms and polyps									
Kiesslich et al. 2004 [82]	Germany	42 patients 390 lesions	64.2	n/a 97.4	n/a 99.4	n/a n/a	n/a n/a	n/a 99.2	CLE enables virtual histology of neoplastic changes with high accuracy, optimizing diagnosis during colonoscopy.
Odagi et al. 2007 [83]	Japan	45 patients	63	n/a	n/a	n/a	n/a	n/a	CEM provides endoscopists with a valuable new diagnostic tool, for observing tissue in situ at the histopathological level, allowing evaluation of physiological function during endoscopic examination.
Wang et al. 2007 [84]	USA	54 patients	n/a	91	87	n/a	n/a	89	CLE provides in vivo real time pathological interpretation of tissue.
Buchner et al. 2010 [85]	USA	75 patients 119 lesions	73	n/a 88	n/a 76	n/a n/a	n/a n/a	n/a n/a	CLE demonstrates higher sensitivity than chromoendoscopy with similar specificity in differentiating colorectal polyps.
De Palma et al. 2010 [86]	Italy	20 patients 32 lesions	62.5	n/a 100	n/a 84.6	n/a 90.5	n/a 100	n/a 92.3	pCLE permits high-quality imaging enabling prediction of intraepithelial neoplasia with high level of accuracy.
Gómez et al. 2010 [87]	USA, Netherlands, and Germany	53 patients 75 lesions	n/a	n/a 76	n/a 72	n/a n/a	n/a n/a	n/a 75	An international collaboration group had moderate to good interobserver agreement using a pCLE system to predict neoplasia.
Sanduleanu et al. 2010 [88]	Netherlands	72 patients 116 lesions	72	n/a 97.3	n/a 92.8	n/a n/a	n/a n/a	n/a 95.7	C-CLE accurately discriminates adenomatous from nonadenomatous colorectal polyps and enables evaluation of degree of dysplasia during ongoing endoscopy.
Xie et al. 2011 [89]	China	115 patients 115 lesions	51.6	n/a 93.9	n/a 95.9	n/a 96.9	n/a 92.2	n/a n/a	Endoscope integrated CLE with fluorescein staining may reliably assist in the real-time identification of colonic adenomas.
André et al. 2012 [90]	USA, France	71 patients 135 lesions	75	n/a 91.4	n/a 85.7	n/a n/a	n/a n/a	n/a 89.6	The proposed software for automated classification of pCLE videos of colonic polyps achieves high performance, comparable to that of offline diagnosis of pCLE videos established by expert endoscopists.
Cârţână et al. 2012 [91]	Romania	4 patients	n/a	n/a	n/a	n/a	n/a	n/a	Imaging of blood vessels with CLE is feasible in normal and tumor colorectal tissue by using fluorescently labeled antibodies targeted against an endothelial marker. The method could be translated into the clinical setting for monitoring of antiangiogenic therapy.
Coron et al. 2012 [92]	France USA	16 patients 13 lesions	62	n/a	n/a	n/a	n/a	n/a	Standard colonic biopsies obtained during CLE retain fluorescein, show excellent delineation of mucosal structures without additional staining, allow the evaluation of mucosal microvasculature and vascular permeability, and are suitable for immunostaining.
Kuiper et al. 2012 [93]	Netherlands	64 patients 154 lesions	59	n/a 57.1	n/a 71	n/a n/a	n/a n/a	n/a 66.7	The majority of p-CLE videos demonstrated insufficient quality in more than half of the time recorded. Post hoc accuracy of p-CLE was significantly lower in comparison with real-time accuracy of CLE and NBI.
Mascolo et al. 2012 [94]	Italy	22 patients	61.6	n/a	n/a	n/a	n/a	n/a	By p-CLE, it is possible to identify specific crypt architecture modifications associated with changes in cellular infiltration and vessels architecture, highlighting a good correspondence between p-CLE features and histology.
Shahid et al. 2012 [95]	USA	74 patients 154 lesions	69	n/a 81	n/a 76	n/a 78	n/a 79	n/a 79	Real-time and offline interpretations of p-CLE images are moderately accurate. Real-time interpretation is slightly less accurate than offline diagnosis.
Shahid et al. 2012 [96]	USA	65 patients 130 lesions	69	n/a 86	n/a 78	n/a n/a	n/a n/a	n/a 82	p-CLE demonstrated higher sensitivity in predicting histology of small polyps compared with NBI, whereas NBI had higher specificity. When used in combination, the accuracy of pCLE and NBI was extremely high, approaching the accuracy of histopathology.
Shahid et al. 2012 [97]	USA, Netherlands	92 patients 129 lesions	70	n/a 97	n/a 77	n/a 55	n/a 99	n/a 81	Confocal endomicroscopy increases the sensitivity for detecting residual neoplasia after colorectal EMR compared with endoscopy alone. In combination with virtual chromoendoscopy, the accuracy is extremely high, and sensitivity approaches that of histopathology.
Gómez et al. 2013 [98]	USA	85 patients 127 lesions	72	n/a 43.4	n/a 70.6	n/a 18.6	n/a 89	n/a n/a	The attempt at creating classification criteria for probe-based CLE did not consistently distinguish advanced from nonadvanced adenomas and, therefore, is not useful in applying a “diagnose, resect, and discard” strategy.
Liu et al. 2013 [99]	China	71 patients 166 lesions	57.6	n/a 97.1	n/a 96.9	n/a n/a	n/a n/a	n/a 97.6	CLE has the potential to enable an immediate diagnosis of CRC and the degree of differentiation of CRC during ongoing endoscopy in vivo.
Liu et al. 2013 [100]	China	37 patients 37 lesions	70	n/a	n/a	n/a	n/a	n/a	CLE could be used in molecular imaging with specific targeting of EGFR in colorectal neoplasia.
Ciocâlteu et al. 2014 [101]	Romania	5 patients	n/a	n/a	n/a	n/a	n/a	n/a	Differences in vessels morphology with CLE are useful for identifying patients who might benefit from neoadjuvant angiogenetic therapy.
Yuan et al. 2014 [102]	China	39 patients 50 lesions	52	n/a 79	n/a 83	n/a n/a	n/a n/a	n/a 81	Three different confocal laser endomicroscopy (CLE) diagnostic systems including Maiz, Sanduleanu, and Qilu for the prediction of colorectal hyperplastic polyp or adenoma have a high accuracy, sensitivity, and specificity.

Graft versus Host Disease									
Bojarski et al. 2009 [103]	Germany	35 patients	n/a	74	100	n/a	n/a	n/a	CLE provides rapid diagnosis of acute intestinal GVHD with high accuracy while performing endoscopy.

Infectious colitis									
Neumann et al. 2013 [104]	Germany	10 patients	72.5	88.9	97.2	80	98.6	96.25	CLE has the potential for in vivo diagnosis of CDI associated colitis. In addition, CLE allowed the detection of intramucosal bacteria in vivo.

Irritable bowel syndrome									
Turcotte et al. 2013 [105]	Canada	34 patients	45.1	62	89	83	73	n/a	As a result of CLE analysis, IBS patients have significantly more epithelial gaps in their small intestine compared with healthy controls, which may be a cause of altered intestinal permeability observed in IBS.
Fritscher-Ravens et al. 2014 [106]	Germany USA UK	36 patients	44.6	n/a	n/a	n/a	n/a	n/a	Based on CLE analysis of IBS patients with a suspected food intolerance, exposure to candidate food antigens caused immediate breaks, increased intervillous spaces, and increased IELs in the intestinal mucosa.

CLE stands for confocal laser endomicroscopy; p-CLE, probe-based confocal laser endomicroscopy; c-CLE, colon probe-based confocal laser endomicroscopy; UC, Ulcerative Colitis; IBD, inflammatory bowel disease; CD, Crohn's disease; NBI, narrow binding imaging; GVHD, Graft versus Host Disease; PPV, positive predictive value; NPV, negative predictive value; and BE, Barrett's esophagus.

**Table 4:** Summary of the studies evaluating CLE in biliary disease screening and diagnosis (all “per patient” analysis).

Author and year	Country	*N*	Mean age (yr)	Sensitivity (%)	Specificity (%)	PPV (%)	NPV (%)	Accuracy (%)	Main findings
Meining et al. 2008 [107]	Germany	14 patients	61.3	83	88	/	/	86	p-CLE considerably increases sensitivity for the detection of biliary neoplasia and therefore represents a promising diagnostic approach.
Giovannini et al. 2011 [108]	France	37 patients	62.3	83	75	n/a	n/a	86	Intrabiliary pCLE is feasible.
Meining et al. 2011 [109]	Germany USA	89 patients	62.8	98	67	71	97	81	p-CLE provides reliable microscopic examination and has excellent sensitivity and NPV with significantly higher accuracy of ERCP and pCLE compared with ERCP with tissue acquisition.
Meining et al. 2012 [110]	Germany USA	47 and 42 patients	63.2 63	97	33	80	80	n/a	The Miami Classification enables a structured, uniform, and reproducible description of pancreaticobiliary pCLE.
Shieh et al. 2012 [2]	USA	10 patients	56.9	n/a	n/a	n/a	n/a	n/a	pCLE of the CBD via the GastroFlex^UHD^ miniprobe is feasible and may offer improved image quality over the standard CholangioFlex probe.
Caillol et al. 2013 [111]	France USA	60 patients	62.2	96.3	75.7	76.5	96.2	85	The Paris Classification improve the accuracy of pCLE for diagnosing benign inflammatory strictures.
Caillol et al. 2013 [112]	France	54 patients	66	88	83	n/a	n/a	87	Inflammation from prestenting procedures interferes with pCLE diagnosis of the bile duct lesions.
Heif et al. 2013 [113]	USA	15 patients	54	100	61.1	22.2	100	n/a	pCLE may have a high sensitivity and negative predictive value to exclude neoplasia in patients with PSC and DS.
Caillol et al. 2015 [114]	France	61 patients	67	88	79	92	69	85	The addition of a pCLE procedure in the diagnostic histologic examination of a biliary stricture permits a significant increase in diagnostic reliability and allows for a VPN of 100%.
Slivka et al. 2015 [115]	USA Italy France	112 patients	64.5	89	71	93	82	88	pCLE provided a more accurate and sensitive diagnosis of cholangiocarcinoma compared with tissue sampling alone.

*N* stands for the number of patients enrolled in the study; pCLE, probe-based confocal laser endomicroscopy; CLE, confocal laser endomicroscopy; PPV, positive predictive value; NPV, negative predictive value; PSC, primary sclerosing cholangitis; DS, dominant biliary stricture; and CBD, common bile duct.

**Table 5:** Summary of the studies evaluating CLE in pancreatic disease screening and diagnosis (all “per patient” analysis).

Authors and year	Country	*N*	Mean age (yr)	Sensitivity (%)	Specificity (%)	PPV (%)	NPV (%)	Accuracy (%)	Main findings
Konda et al. 2011 [15]	USA	18 patients	57.9	n/a	n/a	n/a	n/a	n/a	nCLE is the pancreas is technically feasible.
Konda et al. 2013 [14]	USA Germany France	66 patients	63.1	59	100	100	50	71	nCLE has a high specificity in the detection of PCN but it may be limited by a low sensitivity.
Kahaleh et al. 2015 [116]	USA France	18 patients	58.3	n/a	n/a	n/a	n/a	94	CLE is effective in assisting with diagnosis of indeterminate pancreatic duct strictures prior to surgery.
Nakai et al. 2015 [117]	USA	30 patients	72	87	77	100	100	77	The combination of cystoscopy and nCLE of pancreatic cysts appears to have strong concordance with the clinical diagnosis of PCN.
Napoléon et al. 2015 [118]	France	31 patients	57	69	100	100	82	87	The newly developed nCLE criterion seems to be highly specific for the diagnosis of serous cystadenoma.

*N* stands for the number of patients enrolled in the study; nCLE, needle-based confocal laser endomicroscopy; CLE, confocal laser endomicroscopy; PPV, positive predictive value; NPV, negative predictive value; and PCN, pancreatic cystic neoplasms.
