# Supplementary material for: Confocal Laser Endomicroscopy in Gastrointestinal and Pancreatobiliary Diseases: A Systematic Review and Meta-Analysis
Source: Biomed Res Int. 2016 Feb 17;2016:4638683. doi: 10.1155/2016/4638683 (PMC4773527; doi:10.1155/2016/4638683)
Supplement: Supplementary file 1 — The Supplementary Material contains: Figures S1,S2,S3 describing the meta-analysis of studies about H. pylori infection, Celiac disease and pancreatic cyst neoplasms. Table S1 describes the characteristics of different CLE devices. Tables S2, S3 describe the Quality Assessment of the all the studies included in the review based on the Cochrane criteria for randomized clinical trials and the Newcastle-Ottawa Scale (NOS) for nonrandomized studies. [file 4638683.f1.zip › Figure S1.docx]

**Figure S1. “Per patient” meta-analysis for the application of CLE in *H. pylori* infection: a) pooled sensitivity; b) pooled specificity; c) pooled positive likelihood ratio (LR); d) pooled negative LR.**

**a)**

**b)**

**c)**


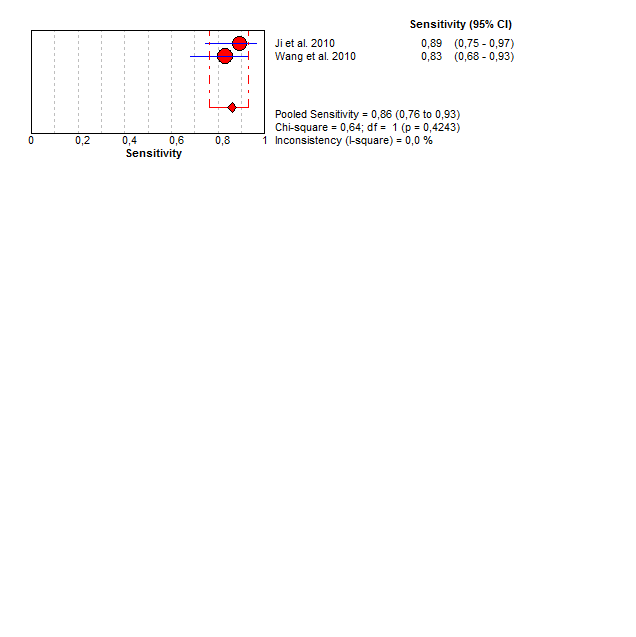

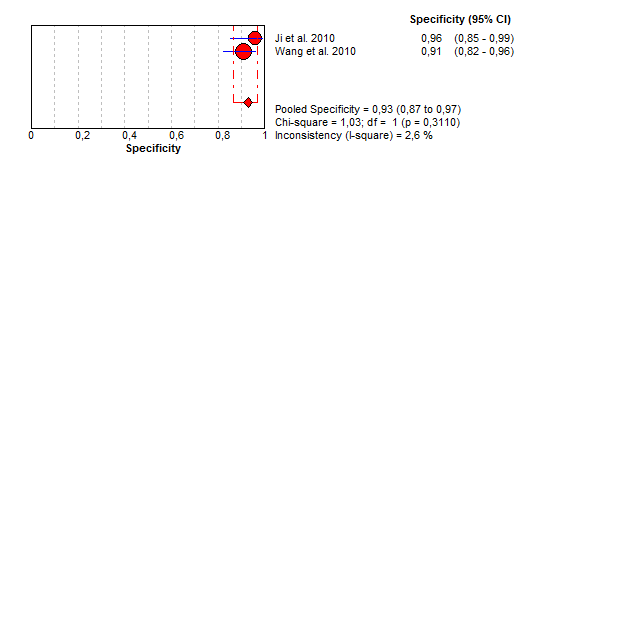

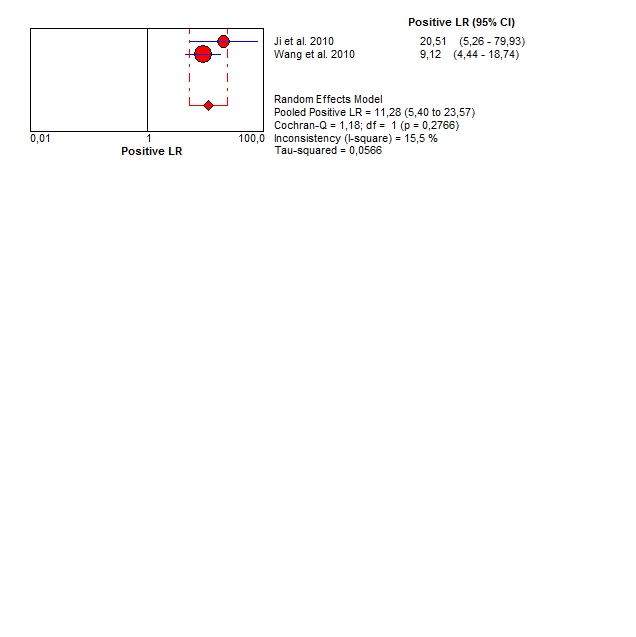

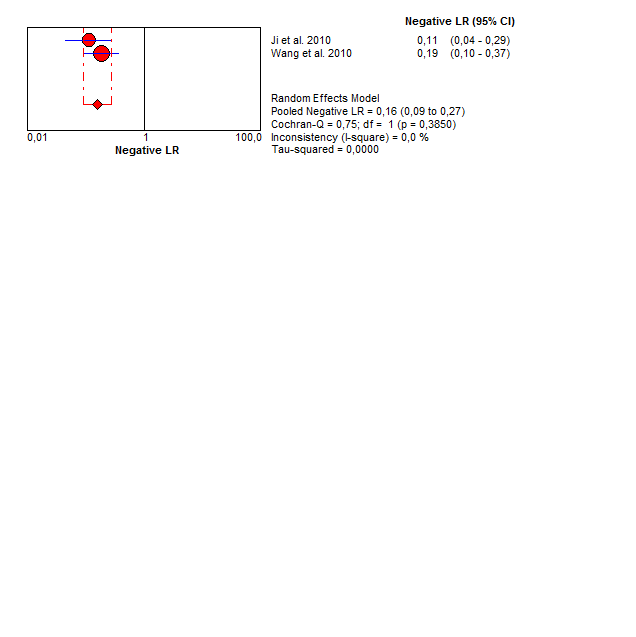


**d)**
