# Supplementary material for: Confocal Laser Endomicroscopy in Gastrointestinal and Pancreatobiliary Diseases: A Systematic Review and Meta-Analysis
Source: Biomed Res Int. 2016 Feb 17;2016:4638683. doi: 10.1155/2016/4638683 (PMC4773527; doi:10.1155/2016/4638683)
Supplement: Supplementary file 1 — The Supplementary Material contains: Figures S1,S2,S3 describing the meta-analysis of studies about H. pylori infection, Celiac disease and pancreatic cyst neoplasms. Table S1 describes the characteristics of different CLE devices. Tables S2, S3 describe the Quality Assessment of the all the studies included in the review based on the Cochrane criteria for randomized clinical trials and the Newcastle-Ottawa Scale (NOS) for nonrandomized studies. [file 4638683.f1.zip › Table S1.docx]

| Confocal endomicroscope | Imaging Depths | Lateral Resolution | Field of view | Compatible channel | Image acquisition  (frames/s) |
| --- | --- | --- | --- | --- | --- |
| e-CLE | 0-250 µm | 0.7 µm | 475 µm | / | 0.8-1.6 |
| GAstroFlex | 70-130 µm | 3.5 µm | 600 µm | 2.8 mm | 12 |
| GAstroFlex^UHD^ | 55-65 µm | 1 µm | 240 µm | 2.8 mm | 12 |
| ColoFlex | 70-130 µm | 3.5 µm | 600 µm | 2.8 mm | 12 |
| ColoFlex^UHD^ | 55-65 µm | 1 µm | 240 µm | 2.8 mm | 12 |
| CholangioFlex | 40-70 µm | 3.5 µm | 325 µm | 1.2 mm | 12 |
| AQ-Flex 19 | 40-60 µm | 3.5 µm | 325 µm | 19 G FNA needle | 12 |

**Table S1.** **Characteristics of different CLE devices.**
