# Supplementary material for: Confocal Laser Endomicroscopy in Gastrointestinal and Pancreatobiliary Diseases: A Systematic Review and Meta-Analysis
Source: Biomed Res Int. 2016 Feb 17;2016:4638683. doi: 10.1155/2016/4638683 (PMC4773527; doi:10.1155/2016/4638683)
Supplement: Supplementary file 1 — The Supplementary Material contains: Figures S1,S2,S3 describing the meta-analysis of studies about H. pylori infection, Celiac disease and pancreatic cyst neoplasms. Table S1 describes the characteristics of different CLE devices. Tables S2, S3 describe the Quality Assessment of the all the studies included in the review based on the Cochrane criteria for randomized clinical trials and the Newcastle-Ottawa Scale (NOS) for nonrandomized studies. [file 4638683.f1.zip › Table S2.docx]

**Table S2. Risk of bias for randomized clinical trials based on the Cochrane criteria.**

| Kiesslich et al 2007 ^[1]^ | 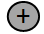 | 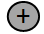 | 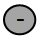 | 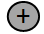 | 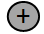 | 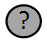 | 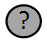 |
| --- | --- | --- | --- | --- | --- | --- | --- |
| Dunbar et al. 2009^[2]^ | 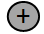 | 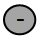 | 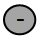 | 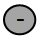 | 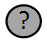 | 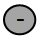 | 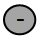 |
| Sharma et al 2011^[3]^ | 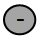 | 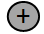 | 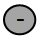 | 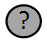 | 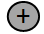 | 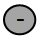 | 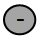 |
| Nguyen et al 2012^[4]^ | 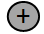 | 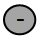 | 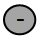 | 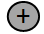 | 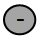 | 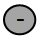 | 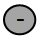 |
| Wallace et al.2012^[5]^ | 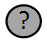 | 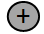 | 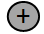 | 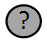 | 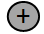 | 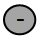 | 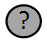 |
| Pittayanon et al 2013^[6]^ | 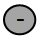 | 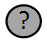 | 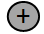 | 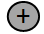 | 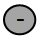 | 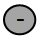 | 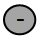 |
| Canto et al. 2014 ^[7]^ | 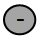 | 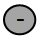 | 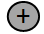 | 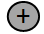 | 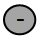 | 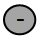 | 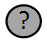 |
| Li et al. 2014^[8]^ | 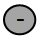 | 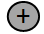 | 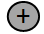 | 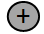 | 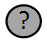 | 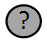 | 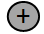 |

Other bias

Selective reporting

Incomplete out come data

Blinding of out come assessment

Blinding of participants and personnel

Other bias

Incomplete outcome data

Random sequence generation

Other bias

Selective reporting

Incomplete outcome data

Blinding of out come assessment

Blinding of participants and personnel

Allocation concealment

Random sequence generation


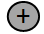
 Low risk of bias
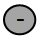
 High risk of bias
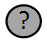
 Unclear risk of bias

**Reference of Table S2**

^[1]^ Kiesslich, R., et al., *Chromoscopy-guided endomicroscopy increases the diagnostic yield of intraepithelial neoplasia in ulcerative colitis.* Gastroenterology, 2007. 132(3): p. 874-82.

^[2]^ Dunbar, K.B., et al., *Confocal laser endomicroscopy in Barrett's esophagus and endoscopically inapparent Barrett's neoplasia: a prospective, randomized, double-blind, controlled, crossover trial.* Gastrointest Endosc, 2009. 70(4): p. 645-54.

^[3]^ Sharma, P., et al., *Real-time increased detection of neoplastic tissue in Barrett's esophagus with probe based confocal laser endomicroscopy: final results of an international multicenter, prospective, randomized, controlled trial.* Gastrointest Endosc, 2011. 74(3): p. 465-72.

^[4]^ Nguyen, V.X., et al., *Confocal endomicroscopy (CEM) improves efficiency of Barrett surveillance.* Interv Gastroenterol, 2012. 2(2): p. 61-65

^[5]^ Wallace, M.B., et al., *Multicenter, randomized, controlled trial of confocal laser endomicroscopy*

*assessment of residual metaplasia after mucosal ablation or resection of GI neoplasia in Barrett's*

*esophagus.* Gastrointest Endosc, 2012. 76(3): p. 539-47 e1.

^[6]^ Pittayanon R., et al., *Flexible spectral imaging color enhancement plus probe-based confocal*

*laser endomicroscopy for gastric intestinal metaplasia detection*, Journal of Gastroenterology and Hepatology 28 (2013) 1004–1009

^[7]^ Canto, M.I., et al., *In vivo endomicroscopy improves detection of Barrett's esophagus-related neoplasia: multicenter international randomized controlled trial (with video).* Gastrointest Endosc, 2014. 79(2): p. 211-.

^[8]^ Li, Z., et al*., Confocal laser endomicroscopy for in vivo detection of gastric intestinal metaplasia: a*

*randomized controlled trial*, Endoscopy 2014; 46: 282–290
