# Supplementary material for: Confocal Laser Endomicroscopy in Gastrointestinal and Pancreatobiliary Diseases: A Systematic Review and Meta-Analysis
Source: Biomed Res Int. 2016 Feb 17;2016:4638683. doi: 10.1155/2016/4638683 (PMC4773527; doi:10.1155/2016/4638683)
Supplement: Supplementary file 1 — The Supplementary Material contains: Figures S1,S2,S3 describing the meta-analysis of studies about H. pylori infection, Celiac disease and pancreatic cyst neoplasms. Table S1 describes the characteristics of different CLE devices. Tables S2, S3 describe the Quality Assessment of the all the studies included in the review based on the Cochrane criteria for randomized clinical trials and the Newcastle-Ottawa Scale (NOS) for nonrandomized studies. [file 4638683.f1.zip › Table S3.docx]

**Table S3. Risk of bias for non-randomized clinical trials based on the *Newcastle-Ottawa Scale*. The study quality was assessed on nine items categorized into three main criteria. A maximum of 9 stars can be assigned to the highest quality. Studies awarded with 7 stars or more were considered at low risk of bias.**

| **Criteria**  **Study** | **Selection** | **Comparability** | **Exposure/ Outcome** | **Overall**  **Score** |
| --- | --- | --- | --- | --- |
| Liu et al. 2008 ^[1]^ | ★★★ | - | ★★ | 5/9 |
| Leong et al. 2008 ^[2]^ | ★★★★ | - | ★★ | 6/9 |
| Watanabe et al. 2008^[3]^ | ★★ | - | ★★ | 4/9 |
| Günther et al. 2010^[4]^ | ★★ | ★ | ★★ | 5/9 |
| Venkatesh et al. 2010^[5]^ | ★★★ | ★★ | ★★ | 7/9 |
| Liu et al. 2011^[6]^ | ★★★ | ★ | ★★ | 6/9 |
| Günther et al. 2011^[7]^ | ★★ | ★ | ★★ | 5/9 |
| Moussata et al. 2011^[8]^ | ★★ | - | ★★ | 4/9 |
| Chu et al. 2012^[9]^ | ★★★ | ★ | ★★ | 6/9 |
| Ji et al. 2012^[10]^ | ★★★★ | ★★ | ★★ | 8/9 |
| Kiesslich et al. 2012^[11]^ | ★★★ | - | ★★ | 5/9 |
| Krauss et al. 2012^[12]^ | ★★ | ★ | ★★ | 5/9 |
| Mascolo et al. 2012^[13]^ | ★★ | ★ | ★★ | 5/9 |
| Neumann et al. 2012^[14]^ | ★★★★ | ★ | ★★ | 7/9 |
| Turcotte et al. 2012^[15]^ | ★★★ | ★ | ★★ | 6/9 |
| Wang et al. 2012^[16]^ | ★★ | ★★ | ★★ | 6/9 |
| Bertani et al. 2013^[17]^ | ★★★★ | ★ | ★★★ | 8/9 |
| Buda et al. 2013^[18]^ | ★★★★ | ★★ | ★★ | 8/9 |
| Caillol et al. 2013^[19]^ | ★★★ | ★ | ★★ | 6/9 |
| Musquer et al. 2013^[20]^ | ★★★ | ★ | ★★ | 6/9 |
| Fritscher-Ravens et al. 2014^[21]^ | ★★ | ★ | ★★ | 5/9 |
| Lim et al. 2014^[22]^ | ★★ | ★ | ★★ | 5/9 |

**Reference of Table S3.**

^[1]^ Liu H., et al., *Confocal endomicroscopy for in vivo detection of microvascular architecture in normal and malignant lesions of upper gastrointestinal tract*, Journal of Gastroenterology and Hepatology 23 (2008) 56–6.

^[2]^ Leong, R.W., et al., *In vivo confocal endomicroscopy in the diagnosis and evaluation of celiac disease.* Gastroenterology, 2008. 135(6): p. 1870-6.

^[3]^ Watanabe, O., et al., *Confocal endomicroscopy in patients with ulcerative colitis.* J Gastroenterol Hepatol, 2008. 23 Suppl 2: p. S286-90.

^[4]^ Günther U. et al., Diagnostic value of confocal endomicroscopy in celiac disease, Endoscopy 2010;42: 197–202.

^[5]^ Venkatesh, K., et al., *Role of confocal endomicroscopy in the diagnosis of celiac disease.* J Pediatr Gastroenterol Nutr, 2010. 51(3): p. 274-9.

^[6]^ Liu J., et al., *Increased epithelial gaps in the small intestines of patients with inflammatory bowel disease: density matters*, Gastrointestinal Endoscopy Volume 73, No. 6 : 2011.

^[7]^ Günther U., et al., *Surveillance colonoscopy in patients with inflammatory bowel disease: comparison of random biopsy vs. targeted biopsy protocols*, Int J Colorectal Dis (2011) 26:667–672

^[8]^ Moussata D., et al., *Confocal laser endomicroscopy is a new imaging modality for recognition of intramucosal bacteria in inflammatory bowel disease in vivo*, Gut 2011;60:26e33.

^[9]^ Chu, C.L., et al., *Microalterations of esophagus in patients with non-erosive reflux disease: in-vivo diagnosis by confocal laser endomicroscopy and its relationship with gastroesophageal reflux.* Am J Gastroenterol, 2012. 107(6): p. 864-74.

^[10]^ Ji R., et al., *Mucosal barrier defects in gastric intestinal metaplasia: in vivo evaluation by confocal endomicroscopy*, Gastrointestinal Endoscopy Volume 75, No. 5 : 2012.

^[11]^ Kiesslich R., et al., *Local barrier dysfunction identified by confocal laser endomicroscopy predicts relapse in inflammatory bowel disease*, Gut 2012;61:1146e1153.

^[12]^ Krauss, E., et al., *Characterization of lymphoid follicles with red ring signs as first manifestation of early Crohn's disease by conventional histopathology and confocal laser endomicroscopy.* Int J Clin Exp Pathol, 2012. 5(5): p. 411-21.

^[13]^ Mascolo, M., et al., *Probe-based confocal laser endomicroscopy evaluation of colon preneoplastic lesions, with particular attention to the aberrant crypt foci, and comparative assessment with histological features obtained by conventional endoscopy.* Gastroenterol Res Pract, 2012. 2012: p. 645173.

^[14]^ Neumann H., et al., *Assessment of Crohn’s Disease Activity by Confocal Laser Endomicroscopy, Inflamm Bowel Dis,* Volume 18, Number 12, December 2012.

^[15]^ Turcotte, J.F., et al., *Increased epithelial gaps in the small intestine are predictive of hospitalization and surgery in patients with inflammatory bowel disease.* Clin Transl Gastroenterol, 2012.

^[16]^ Wang S-F., et al., *Diagnosis of gastric intraepithelial neoplasia by narrowband imaging and confocal laser endomicroscopy*, World J Gastroenterol 2012 September 14; 18(34): 4771-4780.

^[17]^ Bertani, H., et al., *Improved detection of incident dysplasia by probe-based confocal laser endomicroscopy in a Barrett's esophagus surveillance program.* Dig Dis Sci, 2013. 58(1): p. 188-93.

^[18] ]^ Buda, A., et al., *Confocal laser endomicroscopy for prediction of disease relapse in ulcerative colitis: a pilot study.* J Crohns Colitis, 2014. 8(4): p. 304-11.

^[19]^ Caillol, F., et al., *Endomicroscopy in bile duct: Inflammation interferes with pCLE applied in the bile duct: A prospective study of 54 patients.* United European Gastroenterol J, 2013. 1(2): p. 120-7.

^[20]^ Musquer, N., et al., *Probe-based confocal laser endomicroscopy: a new method for quantitative analysis of pit structure in healthy and Crohn's disease patients.* Dig Liver Dis, 2013. 45(6): p. 487-92.

^[21]^ Fritscher-Ravens, A., et al., *Confocal endomicroscopy shows food-associated changes in the intestinal mucosa of patients with irritable bowel syndrome.* Gastroenterology, 2014. 147(5): p. 1012-20 e4.

^[22]^ Lim L. G., et al.,*Confocal endomicroscopy identifies loss of local barrier function in the duodenum of patients with Crohn'sdisease and ulcerative colitis*, [Inflamm Bowel Dis.](http://www.ncbi.nlm.nih.gov/pubmed/?term=Confocal+endomicroscopy+identifies+loss+of+local+barrier+function+in+the+duodenum+of+patients+with+Crohn%27s+disease+and+ulcerative+colitis) 2014 May;20(5):892-900.
